# Forage Crop Research in the Modern Age

**DOI:** 10.1002/advs.202415631

**Published:** 2025-06-30

**Authors:** Qikun Liu, Gai Huang, Zhiqiang Zhang, Zhelong Lin, Xian Deng, Xueming Dong, Zhipeng Liu, Zan Wang, Yingjun Zhang, Hai‐chun Jing, Kang Chong, Xiaofeng Cao

**Affiliations:** ^1^ State Key Laboratory of Gene Function and Modulation Research, Beijing Advanced Center of RNA Biology (BEACON) School of Advanced Agricultural Sciences, Peking University Beijing 100871 China; ^2^ Laboratory of Advanced Breeding Technologies, Institute of Genetics and Developmental Biology Chinese Academy of Sciences Beijing 100101 China; ^3^ Key Laboratory of Grassland Resources of the Ministry of Education, Technology Engineering Center of Drought and Cold‐Resistant Grass Breeding in the North of the National Forestry and Grassland Administration, College of Grassland Science Inner Mongolia Agricultural University Hohhot 010010 China; ^4^ State Key Laboratory of Herbage Improvement and Grassland Agro‐ecosystems College of Pastoral Agriculture Science and Technology Lanzhou University Lanzhou 730020 China; ^5^ College of Grassland Science and Technology China Agricultural University Beijing 100193 China; ^6^ Engineering Laboratory for Grass‐Based Livestock Husbandry Institute of Botany Chinese Academy of Sciences Beijing 100093 China; ^7^ University of Chinese Academy of Sciences Beijing 100049 China; ^8^ Key Laboratory of Plant Molecular Physiology Institute of Botany Chinese Academy of Sciences Beijing 100093 China

**Keywords:** alfalfa, crop improvements, forage crops, plant breeding, ploidy breeding

## Abstract

Forage crops not only provide food for livestock to meet the growing demands of the global population but are also essential for sustainable agricultural systems by rehabilitating infertile and marginal lands. A wide diversity of plant species is cultivated as forage crops, many of which possess complex genetic backgrounds, making them more challenging to improve compared to model plants and staple crops. Recent advancements in molecular biology, sequencing technologies, and genomic analysis tools have opened new avenues for the improvement of forage crops. This review provides a comprehensive examination of modern forage breeding, covering the primary types of forage, their characteristics, key functional genes utilized for enhancing forage traits, advanced breeding technologies, and the challenges and future directions in this field. By integrating the latest research and technological developments, this review aims to contribute to the advancement of forage breeding strategies that can meet the increasing global demand for sustainable and high‐quality forage resources.

## Graphical abstract

Forage crops are increasingly vital to sustainable agriculture, yet urgent demands for transformative innovations create unprecedented opportunities and challenges. This review synthesizes the latest advances in forage research, spotlighting cutting‐edge breeding technologies and their transformative potential. Emerging trends and persistent knowledge gaps are analyzed, offering actionable insights to guide researchers and policymakers in advancing this critical field.

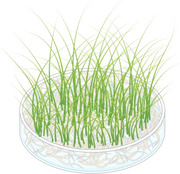

## 1 Introduction

Forage crops are a diverse group of plant species that provide essential feed for livestock. They play a crucial role in sustainable agriculture by enhancing soil fertility, sequestering carbon, reducing soil erosion, and supporting biodiversity. In recent years, forage crops have garnered increasing public attention due to their critical role in meeting the growing demand for livestock‐derived food resources, which are essential for improving human living standards.^[^ ^1^ ^]^ However, forage crops are generally less domesticated. Their outstanding features, such as polyploidy, out‐crossing, and heterogeneous, complex genetic backgrounds, present significant challenges for improving traits like environmental resilience, biomass yield, and forage quality.

Despite these challenges, advances in technology are providing new tools for forage crop breeders. Significant efforts and progress have been made in forage crop research. This review offers a comprehensive exploration of the major types of forage crops cultivated worldwide. We discuss key resources, potential strategies, and emerging technologies that may shape the future of forage crop development. Our goal is to provide readers from diverse backgrounds with a foundational framework of the forage research field. We hope this review not only serves as an accessible entry point for newcomers but also assists readers with specialized expertise in gaining deeper technical insights. Additionally, we recommend seminal reviews recently published by other researchers to facilitate further exploration.^[^ ^2^ ^]^

## 2 Major Classes of Forage Crops

### 2.1 Legume Family

Leguminous forages hold great value in agriculture due to their unique ability to fix atmospheric nitrogen. This process not only enriches their protein content, making them highly nutritious for livestock but also significantly enhances soil fertility while reducing the need for synthetic fertilizers. These features make them essential to sustainable agricultural practices.

**Figure 1:**
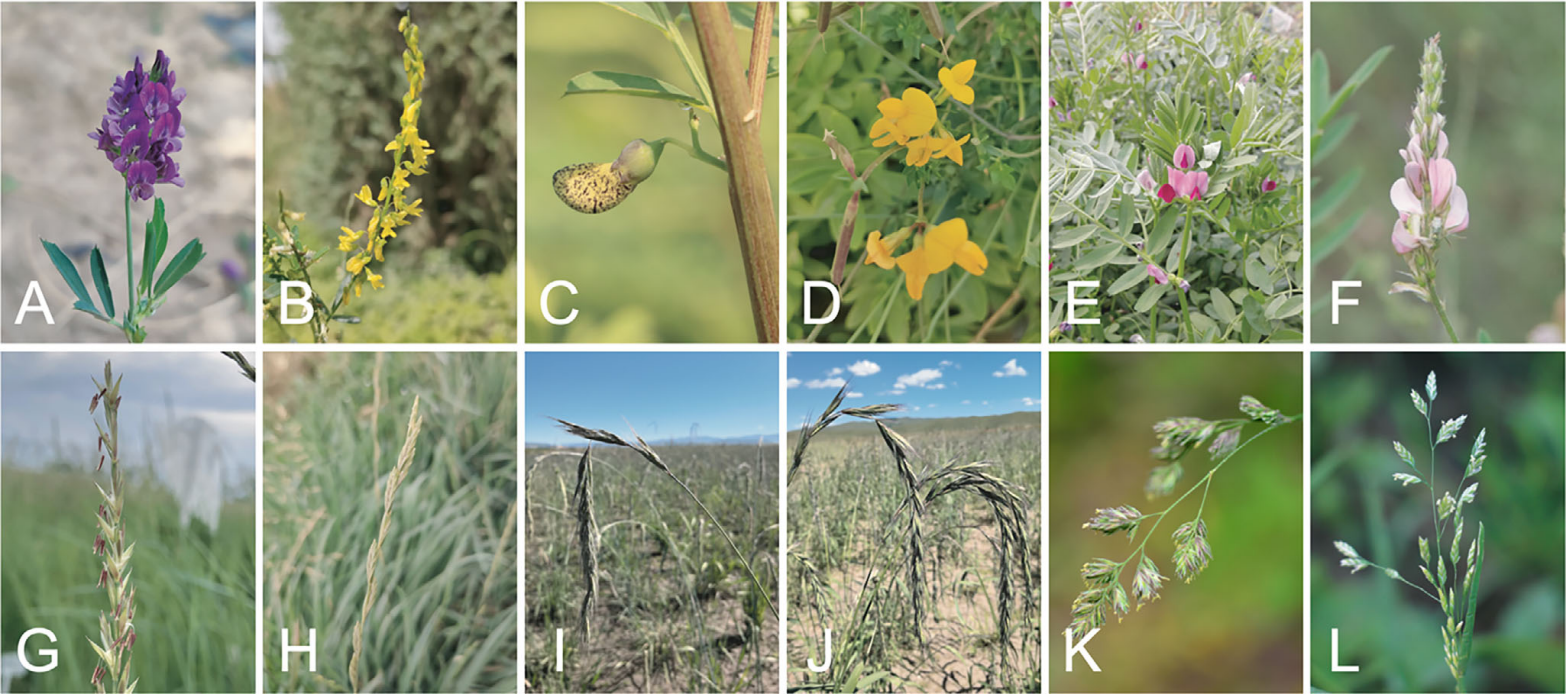
Representative pictures of some major forage crops. A) *Medicago sativa* B) *Melilotus officinalis* C) *Sesbania cannabina* D) *Lotus corniculatus* E) *Vicia sativa* F) *Onobrychis viciifolia* G) *Leymus chinensis* H) *Lolium perenne* I) *Elymus dahuricus* J) *Elymus sibiricus* K) *Dactylis glomerata* L) *Poa annua*.

Among the most widely cultivated forage legumes is *Medicago sativa* (*M. sativa;* alfalfa), often referred to as the “Queen of Forage.” (**Figure** **1**) Alfalfa is renowned for its high protein content and digestibility, making it an excellent forage crop for livestock. Alfalfa originates from Asia Minor/Caucasia and Central Asia but is now cultivated globally, covering ≈45 million hectares (**Table** **1**).^[^ ^3^ ^]^ Cultivated alfalfa is an outcrossing perennial autotetraploid (2*n* = 4× = 32), with its gene pool contributed by several diploid and tetraploid subspecies under the *M. sativa* and *Medicago falcata* taxa.^[^ ^4^ ^]^ Transgenic varieties with glyphosate resistance and low‐lignin content are commercially available.

**Table 1:** Geographic distribution and characteristics of some major forage crops.

Species	Origin/distribution	Life style/reproduction style	Global cultivation area [M ha]
*Medicago sativa* (Alfalfa or lucerne)	Eastern Turkey, Central Iran / Worldwide	Perennial / Cross‐Pollination	45
*Trifolium repens* (White clover)	Mediterranean, West Asia the Atlantic coast of Western Europe / New Zealand, Northwestern Europe, North America	Perennial / Cross‐Pollination	4
*Melilotus officinalis* (Yellow sweet clover)	Temperate Europe, the Mediterranean, subtropical Asia, and northeastern Africa / Temperate Europe, the Mediterranean, subtropical Asia	Annual / Cross‐Pollination	Unknown
*Sesbania cannabina* (Yellow pea bush)	South Pacific Islands, Indonesia, Papua New Guinea / Africa, Asia, Australia	Annual / Self‐Pollination	Unknown
*Onobrychis viciifolia* (Sainfoin)	Asia / Europe, Asia	Perennial / Cross‐Pollination	Unknown
*Vicia sativa* (Common vetch)	Mediterranean and Irano‐Turanian regions / Asia, Europe, and North America	Annual / Self‐Pollination	0.54
*Lotus corniculatus* (Birdfoot trefoil)	East and Central Asia, warm areas of Eurasia / Africa, Asia, Europe	Perennial / Cross‐Pollination	4.62
*Leymus chinensis* (Sheepgrass)	Eastern Eurasian Steppe / Eurasian Steppe, North Korea to Mongolia, northern China	Perennial / Cross‐Pollination	4.2
*Lolium perenne* (Perennial ryegrass)	The Mediterranean basin, Europe, temperate Asia, and North Africa / Africa, Australia, South America	Perennial / Cross‐Pollination	Unknown
*Elymus dahuricus* (Dahurian wildrye)	Central or eastern Asia and the Himalayas / Central Asia, Russia, Mongolia	Perennial / Cross‐Pollination	Unknown
*Elymus sibiricus* (Siberian wildrye)	Northern Asia / Sweden, northern Asia, North America	Perennial / Cross‐Pollination	Unknown
*Dactylis glomerata* (Orchardgrass)	Eurasia and northern Africa / Europe, the Mediterranean basin, West and Central Asia	Perennial / Cross‐Pollination	Unknown
*Pennisetum glaucum* (Ornamental millet)	Western Sahara, Northwest Africa / Japan, India, Korea	Annual / Cross‐Pollination	31.2
*Poa annua* (annual bluegrass)	Europe / North America, Europe, Asia, Australia, and New Zealand	Annual / Cross‐Pollination	Unknown
*× Triticosecale* Wittmack (Triticale)	Europe / Europe (Poland, Germany, Belarus, and France), Russia, China	Annual / Self‐Pollination	4
*Avena sativa* (Oat)	Turkey, Iran, Iraq /World wide	Annual / Self‐Pollination	8

*Trifolium repens* (*T. repens*; white clover) is an allotetraploid perennial forage crop (2*n* = 4× = 32), native to the Mediterranean, West Asia, and the Atlantic coast of Western Europe.^[^ ^5^ ^]^ It spreads through stolons, forming a network of interconnected plants that are resistant to cold, drought, and trampling.^[^ ^6^ ^]^ Studies suggest that allotetraploid white clover originated from interspecific hybridization between two diploid ancestors, the alpine species *T. pallescens* (2*n* = 2× = 16) and the coastal species *T. occidentale* (2*n* = 2× = 16).^[^ ^7^ ^]^ Since these progenitor species occupy distinct habitats, it is believed that this hybridization occurred during the last glaciation when the species coexisted in glacial refugia. The maintenance of subgenome‐specific gene expression may have also facilitated the niche expansion of white clover after the last glaciation.^[^ ^5b^ ^]^ Today, white clover is cultivated on ≈4 million hectares globally, with ≈2200 varieties developed worldwide.

*Melilotus officinalis* (*M. officinalis;* yellow sweet clover) is a highly productive legume forage, known for its superior capacity to support nitrogen fixation and high dry matter yield.^[^ ^8^ ^]^ This diploid forage species (2*n* = 2× = 16), which demonstrates strong tolerance to saline soils and extreme weather conditions, is widespread across the Eurasian continent.^[^ ^9^ ^]^ In addition to being used as green manure and in crop rotation, *Melilotus* species, including *M. officinalis* and its close relative *Melilotus albus*, are valued for their medicinal properties due to their abundance of pharmacologically active coumarins.^[^ ^10^ ^]^

*Sesbania cannabina* (yellow pea bush), native to the South Pacific Islands and Southeast Asia, is widely cultivated across Africa, Asia, and Australia. *S. cannabina* is especially valued for its high biomass production and adaptability to saline–alkaline soils.^[^ ^11^ ^]^ Due to these traits, it is increasingly recognized as a valuable resource for improving marginal lands.^[^ ^12^ ^]^ Its strong resistance to salt and alkali is linked to a unique community of nodulating rhizobia.^[^ ^13^ ^]^ Recently, the telomere‐to‐telomere genome assembly of this allotetraploid legume (2*n *= 4× = 24) was released,^[^ ^14^ ^]^ making it one of the most completely assembled genomes among leguminous forages to date.

In addition to the species mentioned above, several other notable forage legumes offer valuable traits such as high protein content, suitability for grazing, and stress resistance. These include *Onobrychis viciifolia* (sainfoin), *Vicia sativa* (common vetch), *Lotus corniculatus* (birdsfoot trefoil), and *Stylosanthes guianensis* (Stylo).^[^ ^15^ ^]^ *S. guianensis* (2*n* = 2× = 20) is a tropical legume native to South America and is particularly well‐adapted to the drought conditions and low‐phosphate, acidic soils that are typical of tropical regions. Its exceptional tolerance to low phosphate soil is believed to be linked to its high‐acid phosphatase activity.^[^ ^16^ ^]^ Due to these adaptive traits, *S. guianensis* has been introduced to and is now widely cultivated in various tropical regions across Africa, Southeast Asia, and the Hainan Island of China, making it a valuable forage crop in these regions.^[^ ^17^ ^]^

### 2.2 Poaceae Family

Unlike leguminous forage crops, Poaceae grasses do not support symbiotic nitrogen fixation. They are typically characterized by fibrous root systems that strengthen soil structure and prevent erosion. These grasses excel in rapid growth and thrive in various climates and soil types, providing continuous ground cover and energy‐rich feed for livestock. Their ability to withstand grazing and trampling also makes them ideal for pasture management.

*Leymus chinensis* (sheepgrass or Chinese wildrye) is one of the dominant grass species across the Eurasian Steppe and is a crucial pasture grass known for its abundant foliage and ability to endure harsh environmental conditions.^[^ ^18^ ^]^ Its allotetraploid genome (2*n* = 4× = 28) consists of two subgenomes (Ns‐ and Xm‐), which are estimated to have diverged ≈16.79 million years ago.^[^ ^19^ ^]^ *L. chinensis* has also gained attention as an ideal forage crop for marginal lands with relatively high salinity levels and frequent freezing winters.^[^ ^20^ ^]^ However, breeding and improving *L. chinensis* have long been hindered by its complex genetic background, low seed germination rates, and the lack of a genetic transformation system.^[^ ^21^ ^]^ The recent availability of high‐quality genomic resources, the development of SSR markers, and advancements in genome editing platforms have opened new avenues for basic research and breeding of *L. chinensis*.^[^ ^19^, ^22^ ^]^

The genus *Lolium* includes several species of significant agricultural importance, such as *Lolium perenne* (perennial ryegrass, 2*n* = 2× = 14), *Lolium multiflorum* (Italian ryegrass, 2*n* = 2× = 14), and *Lolium rigidum* (annual ryegrass, 2*n* = 2× = 14). *L. perenne* and *L. multiflorum* are extensively used for pastures, turf, and livestock feed, whereas *L. rigidum* is considered a major weed in cropping systems.^[^ ^23^ ^]^ As a result, the physiological traits of *Lolium* species, such as seed germination, have evolved in response to agricultural practices, including weed management.^[^ ^24^ ^]^ Both *L. multiflorum* and *L. rigidum* typically exhibit annual growth patterns and are better adapted to warmer climates. However, they are prone to summer depression and have low cold tolerance.^[^ ^25^ ^]^

*Festuca* species, such as *F. arundinacea* (2*n* = 6× = 42) and *F. pratensis* (2*n* = 2× = 14), are closely related to *Lolium*. These species are more resilient under extreme weather conditions and show stronger resistance to biotic stresses. As a result, *Festuca* species are often used in interspecific crosses with *Lolium* species to produce hybrids with superior agricultural traits.^[^ ^26^ ^]^ These hybrids, known as *Festulolium*, combine the high yield and forage quality of *Lolium* with the stress resistance of *Festuca*, making them more sustainable and productive forage crops, especially in challenging environments.

*Elymus sibiricus* (Siberian wildrye) and *Elymus dahuricus (*Dahurian wildrye), which originate from Central or Eastern Asia and the Himalayas, are notable for their adaptability to high altitudes and their resistance to drought, cold, and wind erosion.^[^ ^27^ ^]^ These *Elymus* species belong to the Triticeae tribe, which consists of ≈350–500 species,^[^ ^28^ ^]^ including many important forage crops such as ×*Triticosecale* Wittmack (triticale) and *Avena sativa* (oat), as well as cereal crops such as *Triticum aestivum* (wheat), *Hordeum vulgare* (barley), and *Secale cereale* (rye). As a result, *Elymus* species are not only forage crops but also an essential gene pool for improving cereal crops.^[^ ^29^ ^]^

The allotetraploid genome of *E. sibiricus* (2*n *= 4× = 28) is composed of St‐ and H‐subgenomes contributed by the genera *Pseudoroegneria* and *Hordeum*, respectively.^[^ ^30^ ^]^ The allohexaploid genome of *E. dahuricus* (2*n* = 6× = 42) includes the St‐, H‐, and Y‐subgenomes; however, the ancient donor of the Y‐subgenome remains uncharacterized.^[^ ^29^, ^30^ ^]^

The *Dactylis* genus contains a single species, *Dactylis glomerata*, commonly known as orchardgrass or cocksfoot. This perennial grass is an excellent cool‐season forage and is widely cultivated across temperate regions in the Northern Hemisphere. Its seed sales place *Dactylis* among the most prominent forage crop genera, alongside *Lolium*, *Festuca*, and *Phleum*. *D. glomerata* exhibits infraspecific polyploidy, with diploid (2*n* = 2× = 14), tetraploid (2*n* = 4× = 28), and in rare instances, hexaploidy (2*n* = 6× = 42) subspecies coexisting and forming a monophyletic group.^[^ ^31^ ^]^

Molecular phylogenetic analyses of different *D. glomerata* subspecies suggest that the diploid subspecies originated in Central Asia and spread across broad regions of the Eurasian steppe before the last glaciation.^[^ ^32^ ^]^ The tetraploid subspecies likely evolved more recently, and its sympatric distribution with the diploid progenitor may have facilitated hybrid vigor between populations.^[^ ^32^ ^]^ Hexaploid subspecies have also been found, though they are rare. Due to the complex taxonomic structure of the *Dactylis* genus, numerous studies have been conducted to clarify the phylogeny of its germplasms, utilizing resources such as microsatellite (SSR) markers and chloroplast genome sequences.^[^ ^33^ ^]^

× *Triticosecale* Wittmack (Triticale), a synthetic hybrid of wheat (*Triticum* spp.) and rye (*Secale cereale* L.) exhibits a range of ploidy levels, from tetraploid (2*n* = 2× = 14) to octoploid (2*n* = 8× = 56). Among these, the hexaploid type (2*n* = 6× = 42) is the most widely cultivated due to its superior adaptability, genomic stability, and productivity. As a dual‐purpose crop, triticale is well‐suited for both forage and grain production.^[^ ^34^ ^]^ When used as a forage crop, triticale demonstrates higher dry matter yield and protein content compared to wheat, while matching rye's nitrogen capture efficiency.^[^ ^35^ ^]^ Additionally, triticale is amenable to both pure line selection and hybrid breeding methods, making it a versatile candidate for agricultural improvement.

*Avena sativa* L. (Oat) (2*n* = 6× = 42) is widely grown for both grain and forage purposes due to its good adaptability and high nutritional value. It serves as a key winter forage crop and is cultivated as a versatile crop for grain and forage straw production. In 2019, global production reached ≈25 million tons, with Russia and Canada each contributing 20%.^[^ ^36^ ^]^ The reference genomes of oats and its diploid and tetraploid ancestors have been released,^[^ ^37^ ^]^ enabling rapid and accurate identification of high‐quality genes and laying a solid foundation for molecular breeding in oats.

In addition to the aforementioned forage crops, several plant species serve as major crops for both humans and livestock and are therefore of significant economic and agricultural value. These crops include *Brassica napus* (forage rapeseed), *Glycine max* (forage soybean), *Zea mays* (forage maize), *T. aestivum* (forage wheat), and *Sorghum bicolor* (sweet sorghum) among others. *Poa pratensis* (Kentucky bluegrass) is another multipurpose grass species, which provides erosion control and serves as both turf and forage crops. Certain plant species also emerge as new types of forage crop, such as *Broussonetia papyrifera* (paper mulberry), which is known for its high biomass, drought tolerance, and nutritional value, and is particularly suitable for mixed planting systems.^[^ ^38^ ^]^

### 2.3 Similarities and Differences between Leguminous and Gramineous Forages

Leguminous and gramineous forages are closely related yet distinct. Leguminous forages, like alfalfa, are known for their high protein content, making them ideal for livestock feed, especially in ruminant diets.^[^ ^2a^ ^]^ They thrive in diverse but well‐suited environments with good soil and climate conditions. Moreover, legumes can support nitrogen fixation through symbiotic relationships, benefiting soil fertility.^[^ ^39^ ^]^ *Gramineous* forages, such as ryegrass and corn, are high‐yielding and highly adaptable, growing in various climates.^[^ ^2^, ^40^ ^]^ They are high in fiber, typically used as silage for animals with high energy needs like dairy cows.

Undoubtedly, leguminous and gramineous forages are vital forage resources widely utilized in animal husbandry.^[^ ^41^ ^]^ They provide animals with substantial nutritional value, delivering essential energy and protein. Moreover, both contribute to soil improvement: legumes enhance soil fertility through nitrogen fixation, while grasses promote soil organic matter accumulation due to their high biomass.^[^ ^42^ ^]^ Importantly, they exhibit strong complementarity. The high protein and mineral content of legumes complements the high energy and fiber content of grasses. Mixed planting or combined use can effectively meet animals' nutritional requirements and support their healthy growth.^[^ ^43^ ^]^

In conclusion, each species within the legume and Poaceae families occupies a unique ecological niche. Together, they play a vital role in supporting sustainable agriculture and healthy ecosystems. To meet the growing demand for forage crops, breeders have made constant efforts toward more efficient utilization and improvement of forage crops. As a prerequisite, it is critical to obtain a comprehensive understanding of their genetic backgrounds. In recent years, with rapid advancements in sequencing and bioinformatics technologies, the genomic landscape of many important forage crop species has been unveiled.^[^ ^19^, ^44^ ^]^

## 3 Genomic Resources of Forage Crops

### 3.1 Genome Assembly

To date, the genomes of at least 41 forage crop species have been sequenced (**Table** **2**). Within the Leguminosae family, the genome of *Medicago sativa* (alfalfa) has been assembled and updated multiple times.^[^ ^45^ ^]^ The genome of its close relative, the diploid *M. truncatula*, a model organism for legume biology, has also been assembled and updated.^[^ ^46^ ^]^ Other resolved *Medicago* genomes include those of *M. sativa* ssp*. caerulea*, *M. polymorpha* (burclover), and *M. ruthenica* (wild alfalfa). These studies have provided valuable insights into the genetic basis underlying various important biological processes in legume forages, such as alkaline stress tolerance, secondary metabolite biosynthesis, nodulation, and legume–rhizobial interactions.

**Table 2:** Forage crops with assembled genomes.

Species	Family	Ploidy level	Assembled haploid genome size	Refs.
*Medicago truncatula*	Leguminosae	Diploid / 2*n* = 2× = 16	481.19 Mb	[46, 51]
*Medicago sativa*	Leguminosae	Autotetraploid / 2*n* = 4× = 32	816 Mb	[45]
*Medicago sativa* ssp. *caerulea*	Leguminosae	Diploid / 2*n* = 2× = 16	793.2 Mb	[4, 52]
*Medicago polymorpha*	Leguminosae	Diploid / 2*n* = 2× = 14	457.53 Mb	[53]
*Medicago ruthenica*	Leguminosae	Diploid / 2*n* = 2× = 16	904.13 Mb	[54]
*Sesbania cannabina*	Leguminosae	Allotetraploid / 2*n* = 4× = 24	2.09 Gb	[14]
*Melilotus albus*	Leguminosae	Diploid / 2*n* = 2× = 16	1.05 Gb	[10a]
*Melilotus officinalis*	Leguminosae	Diploid / 2*n* = 2× = 16	1.07 Gb	[9, 55]
*Trifolium repens*	Leguminosae	Allotetraploid / 2*n* = 4× = 32	1.09 Gb	[5, 56]
*Trifolium occidentale*	Leguminosae	Diploid / 2*n* = 2× = 16	436.8 Mb	[5b]
*Trifolium pallescens*	Leguminosae	Diploid / 2*n* = 2× = 16	382.4 Mb	[5b]
*Astragalus sinicus*	Leguminosae	Diploid / 2*n* = 2× = 16	595.52 Mb	[57]
*Vicia sativa*	Leguminosae	Diploid / 2*n* = 2× = 14	1.59 Gb	[58]
*Chenopodium quinoa*	Chenopodiaceae	Allotetraploid / 2*n* = 4× = 36	1.39 Gb	[59]
*Haloxylon ammodendron*	Chenopodiaceae	Diploid / 2*n* = 2× = 18	685.4 Mb	[60]
*Suaeda glauca*	Chenopodiaceae	Diploid / 2*n* = 2× = 18	1.02 Gb	[61]
*Carex parvula*	Cyperaceae	Tetraploid/ 2*n* = 4× = 64	783.49 Mb	[62]
*Kobresia myosuroides*	Cyperaceae	Diploid / 2*n* = 2× = 58	399.9 Mb	[63]
*Kobresia littledalei*	Cyperaceae	Diploid / 2*n* = 2× = 58	373.85 Mb	[64]
*Achnatherum splendens*	Poaceae	Diploid / 2*n* = 2× = 48	1.17 Gb	[65]
*Avena sativa*	Poaceae	Allohexaploid / 2*n* = 6× = 42	10.76 Gb	[37, 47]
*Bromus tectorum*	Poaceae	Diploid / 2*n* = 2× = 14	2.48 Gb	[66]
*Cleistogenes songorica*	Poaceae	Allotetraploid / 2*n* = 4× = 40	540.12 Mb	[67]
*Cynodon dactylon*	Poaceae	Allotetraploid / 2*n* = 4× = 36	604 Mb	[68]
*Cynodon transvaalensis*	Poaceae	Diploid / 2*n* = 2× = 18	423.42 Mb	[69]
*Dactylis glomerata*	Poaceae	Diploid / 2*n* = 2× = 14	1.84 Gb	[70]
*Elymus sibiricus*	Poaceae	Allotetraploid / 2*n * = 4× = 28	6.93 Gb	[30a]
*Leymus chinensis*	Poaceae	Allotetraploid / 2*n* = 4× = 28	7.85 Gb	[19]
*Lolium perenne*	Poaceae	Diploid / 2*n* = 2× = 14	2.55 Gb	[71]
*Miscanthus sinensis*	Poaceae	Diploid / 2*n* = 2× = 38	1.68 Gb	[72]
*Pennisetum alopecuroides*	Poaceae	Diploid / 2*n* = 2× = 18	845.71 Mb	[73]
*Cenchrus americanus*	Poaceae	Diploid / 2*n* = 2× = 14	1.85 Gb	[48, 74]
*Poa annua*	Poaceae	Allotetraploid / 2*n* = 4× = 28	1.78 Gb	[75]
*Puccinellia tenuiflora*	Poaceae	Diploid / 2n = 2× = 14	1.50 Gb	[76]
*Setaria viridis*	Poaceae	Diploid / 2*n* = 2× = 18	395.1 Mb	[77]
*Secale cereale*	Poaceae	Diploid / 2*n* = 2× = 14	7.74 Gb	[78]
*Sorghum bicolor*	Poaceae	Diploid / 2*n* = 2× = 20	730 Mb	[47, 79]
*Thinopyrum elongatum*	Poaceae	Diploid / 2*n* = 2× = 14	4.63 Gb	[80]
*Cenchrus purpureus*	Poaceae	Allotetraploid / 2*n* = 4× = 28	1.97 Gb	[81]
*Paspalum vaginatum*	Poaceae	Diploid / 2*n* = 2× = 20	646.9 Mb	[82]
*Panicum virgatum*	Poaceae	Allotetraploid / 2*n* = 4× = 36	1.13 Gb	[50]

Significant progress has also been made in genomic assemblies for Poaceae forage species, including *L. chinensis*, *S. bicolor*, *A. sativa*, *D. glomerata*, *P. virgatum*, and *E. sibiricus* (Table 2).^[^ ^19^, ^30^, ^37^, ^47^ ^]^ Despite the challenges in generating haplotype‐resolved genome assemblies for many polyploid species, these studies have not only helped uncover the evolutionary origins of various forage crop species, such as *L. chinensis* and *A. sativa*,^[^ ^19^, ^37^, ^47^ ^]^ but also provide valuable genetic resources to accelerate the genetic improvement of forage crops.

### 3.2 Pan‐Genome and Population Genomics

In addition to genomic resources at the individual level, population genomics and pan‐genome analysis have become essential tools in forage crop breeding. A graph‐pangenome has been constructed using ten representative core germplasm samples of *Pennisetum americanum* (pearl millet), revealing the role of the RWP‐RK transcription factor and the endoplasmic reticulum (ER) system in heat tolerance.^[^ ^48^ ^]^ In sorghum, both pan‐genome construction and population genomic studies have been conducted, uncovering important genetic variations underlying sorghum domestication and improvement.^[^ ^49^ ^]^ Genomic resource studies at the population level have also been reported for other forage crop species, such as *Panicum virgatum* (switchgrass) and alfalfa.^[^ ^45^, ^50^ ^]^ Expanding such studies will certainly contribute to a better understanding of the genetic basis underlying forage crop traits, as well as their genetic diversity and evolutionary history.

## 4 Genes Related to Key Traits of Forage Crops

Many forage crop species are polyploid, heterozygous, and have complex genetic backgrounds (Table 2). The absence of pure inbred lines has also hindered the genetic improvement of forage crops. Molecular breeding approaches, particularly the genetic engineering of gene regulators, have demonstrated success in the improvement of staple crops and hold great promise for forage crop breeding. Below, we summarize advancements in gene function studies concerning key forage crop traits, including abiotic and biotic stress resistance, biomass yields, and quality.

### 4.1 Salinity Tolerance

Forage crops often need to be cultivated in less fertile lands, making strong environmental resilience a highly desirable trait. Salt stress is one of the most significant abiotic stresses limiting the productivity and quality of agricultural and forage crops worldwide. Insights gained from studies in Arabidopsis and other model plants have provided valuable information for improving salinity resistance in forage crops (**Table** **3**). For example, the use of the *miR156‐ SPL* (*QUAMOSA PROMOTER BINDING PROTEIN LIKE*) gene regulatory module has been shown to enhance alfalfa's salinity resistance by affecting the expression of genes associated with Na^+^ accumulation, antioxidant accumulation, amino acids biosynthesis, and photosynthesis.^[^ ^83^ ^]^ Other notable genes that enhance forage crop salt tolerance include *MsWRKY11*, *MsWRKY33*, *MsFLS13*, *MsRCI2A/B/C*, and *LcSAMDC1*.^[^ ^84^ ^]^ Population genetics‐based methods such as GWAS have proven to be powerful strategies for identifying salinity resistance‐related genes. Examples include the identification of *MsFTa2* from alfalfa and *AT1* from sorghum.^[^ ^45^, ^85^ ^]^ These discoveries indicate that diverse mechanisms, including the regulation of reactive oxygen species (ROS) distribution in the cytosol, abscisic acid (ABA) hormone responses, and calcium‐signaling pathways, are involved in the response to salinity stress in forage crops.^[^ ^45^, ^85^, ^86^ ^]^

**Table 3:** Functional genes related to abiotic stress resistance.

Type of stress	Gene name	Species	Refs.
Salt	*SPL12*	*Medicago sativa*	[87]
	*WRKY33*	*Medicago sativa*	[88]
	*MsFLS13*	*Medicago sativa*	[89]
	*MsRCI2A*	*Medicago sativa*	[90]
	*MsRCI2B*	*Medicago sativa*	[90]
	*MsRCI2C*	*Medicago sativa*	[90]
	*miR156*	*Medicago sativa*	[83c]
	*WRKY11*	*Glycine max*	[84a]
	*AT1*	*Sorghum bicolor*	[91]
Drought	*MruGSTU39*	*Medicago sativa*	[92]
	*WXP1*	*Medicago sativa*	[93]
	*KCS10*	*Medicago sativa*	[94]
	*MsDIUP1*	*Medicago sativa*	[95]
	*MsNTF2L*	*Medicago sativa*	[95]
	*MicroRNA156*	*Medicago sativa*	[96]
	*MsSPCH*	*Medicago sativa*	[97]
	*MsMYBH*	*Medicago sativa*	[98]
	*Os‐microRNA408*	*Lolium perenne*	[99]
	*LpHUB1*	*Lolium perenne*	[100]
Cold	*MsCML10*	*Medicago sativa*	[101]
	*AIR12*	*Medicago falcata*	[102]
	*MfSAMS1*	*Medicago falcata*	[103]
	*MfERF*	*Medicago falcata*	[104]
	*MfEF2*	*Medicago falcata*	[105]
	*MfGolS1*	*Medicago falcata*	[106]
	*MfAOC2*	*Medicago falcata*	[107]
	*MfTIL1*	*Medicago falcata*	[108]
	*MfPIP2‐7*	*Medicago falcata*	[109]
	*LcFIN1*	*Leymus chinensis*	[110]
	*LcSAMDC1*	*Leymus chinensis*	[111]
Heat	*HSFA3*	*Lolium perenne*	[112]
	*LpNAL*	*Lolium perenne*	[113]
	*LpSGR*	*Lolium perenne*	[114]
	*PpEXP1*	*Poa pratensis*	[115]

### 4.2 Drought Resistance

Drought significantly reduces the productivity of agricultural crops and is considered one of the most extreme climate events for terrestrial life.^[^ ^116^ ^]^ A large number of genes related to drought resistance have been identified in various forage crops. For instance, in perennial ryegrass, the overexpression of *LpHUB1* and *Os‐miR408* has been shown to enhance drought tolerance.^[^ ^117^ ^]^ In alfalfa, ectopic expression of genes such as *MruGSTU39* (a glutathione S transferase),^[^ ^118^ ^]^ *MtWXP1* (a putative AP2 domain‐containing transcription factor),^[^ ^93^ ^]^ *MsKCS10* (a 3‐ketoacyl‐CoA synthase 10),^[^ ^119^ ^]^ *MsDIUP1* (drought‐induced unknown protein 1),^[^ ^120^ ^]^ *MsNTF2L* (nuclear transport factor 2‐like),^[^ ^121^ ^]^ and miR156 have all been shown to improve drought resistance.^[^ ^96^ ^]^ These genes confer enhanced drought tolerance through various mechanisms, such as antioxidant defense, cuticular wax deposition, and ABA signaling.

### 4.3 Cold and Heat Resistance

Extreme temperatures, both cold and heat, significantly impact forage yield and are key factors determining the cultivation zones of various forage crops. While most forage crop species thrive in regions with mild climate conditions, certain species, such as *Medicago sativa* subsp. *falcata* and *Leymus chinensis*, exhibit strong cold resistance and are therefore used to identify genetic resources for improving cold resistance in other forage crops.^[^ ^122^ ^]^ These studies have led to the discovery of several cold‐resistance genes, including *MfAIR12* (Auxin Induced in Root Culture 12), *MfSAMS1* (S‐adenosylmethionine synthetase), and *MfAOC2* (Allene Oxide Cyclase 2).^[^ ^122b,c,g^ ^]^ The identification of these genes suggests the involvement of several core physiological activities during the cold response in forage crops, including the maintenance of ROS homeostasis, jasmonic acid (JA) signaling, and ABA signaling.^[^ ^122^, ^123^ ^]^

Climate change and global warming have a major impact on crop production. For example, high temperatures during the pollination season can lead to severe reductions in seed yield.^[^ ^124^ ^]^ As extreme heat events become more frequent, there is significant interest in deploying heat‐tolerance genes to mitigate the effects of heat stress. However, heat stress resistance genes in forage crop species remain largely uninvestigated. To date, only a small number of genes related to heat stress resistance have been studied in *Pennisetum purpureum* Schumach. (elephant grass), *Poa pratensis*, *Zygophyllum xanthoxylum*, and *Lolium perenne*.^[^ ^125^ ^]^ These genes enhance heat resistance in plants by regulating photosystem stability, nitrogen use efficiency, and peroxidase gene expression.

### 4.4 Resistance to Microbial Pathogens

Forage crop production is threatened by infections and damage caused by fungi, bacteria, viruses, and aerial and soil pests, which negatively impact both the quality and yield of forage. For example, a survey conducted in the United States indicated a 19.3% yield loss of alfalfa due to foliar diseases.^[^ ^126^ ^]^ In the following section, we review the knowledge of functional genes related to resistance against biotic stress in forage crops (**Table** **4**).

**Table 4:** Functional genes related to biotic stress resistance.

Type of pathogen, disease, or insect pest	Gene name	Species	Refs.
Anthracnose	*RCT1*	*Medicago truncatula*	[131]
*Pseudomonas syringae*	*IOMT*	*Medicago sativa*	[132]
fungal wilts	*β‐1,3‐glucanase*	*Solanum melongena*	[133]
powdery mildew	*Pm7*	*Avena sativa*	[134]
	*Pm11*	*Avena sativa*	[135]
	*Pc54*	*Avena sativa*	[129c]
	*Eg‐3*	*Avena sativa*	[136]
Oat crown/stem rust	*Pc91*	*Avena sativa*	[129e]
	*Pc50‐5*	*Avena sativa*	[137]
	*Pg13*	*Avena sativa*	[138]
	*Pc96*	*Avena sativa*	[139]
	*Pc98*	*Avena sativa*	[140]
pea aphid	*AaEβF*	*Medicago sativa*	[141]
Spodoptera	*cryIC*	*Medicago sativa*	[142]

The main types of microbial pathogen infections in grassland plants include powdery mildew, rust, anthracnose, downy mildew, and root rot.^[^ ^127^ ^]^ These diseases not only cause yield loss and decline forage quality but may also lead to long‐term degradation of grasslands. The focus of disease resistance research differs between leguminous and gramineous forages. The former primarily emphasizes resistance to anthracnose, downy fusarium wilt, and root rot, while the latter concentrates on diseases like powdery mildew and rust.

However, the direct characterization of disease‐resistant genes in many forage crops is hindered by several factors, including polyploidy, complex genetic backgrounds, and the lack of inbred lines. One possible strategy is to clone disease‐resistance genes from close relatives of forage crops that have a less complex genetic background and can be manipulated with fewer difficulties. For example, the diploid genome of *M. truncatula* shows a high degree of sequence conservation with the tetraploid genome of *M. sativa* and can be maintained as pure inbred lines through self‐pollination.^[^ ^51a^ ^]^ In one instance, a host resistance (R) gene, *RCT1*, was cloned from *M. truncatula* and shown to confer broad‐spectrum resistance to anthracnose disease in susceptible alfalfa cultivars.^[^ ^128^ ^]^ RCT1 belongs to the Toll/interleukin‐1 receptor/nucleotide‐binding site/leucine‐rich repeat (TIR‐NBS‐LRR) class of plant R genes. It recognizes pathogen signals from Colletotrichum trifolii and triggers immune responses.

Regarding gramineous forages, extensive studies have been conducted in oats to map the positions of genes resistant to powdery mildew, crown rust, and stem rust.^[^ ^129^ ^]^ It would be of great interest to clone and test the efficacy of these oat‐originated genes in conferring disease resistance in other gramineous forages. Since these studies were conducted in different regions, possibly dominated by various pathogen strains, we can expect variations in the efficacy of each gene when challenged by different pathogen strains.^[^ ^130^ ^]^

### 4.5 Resistance to Insects and Herbivores

Insects and herbivores pose significant threats, causing production losses in forage crops. However, the identification and mechanistic studies of insect‐resistant genes in these crops are relatively limited. Comparative studies between insect‐resistant and susceptible alfalfa cultivars, using transcriptome, proteome, and metabolome profiling, have highlighted the contributions of flavonoid biosynthesis and the JA signaling pathway to insect resistance.^[^ ^143^ ^]^ Additionally, novel traits such as the production of insect‐repelling pheromones and insecticidal toxins can be genetically engineered into forage crops to enhance insect resistance.^[^ ^141^, ^144^ ^]^

### 4.6 Biomass Yield

Unlike major crops with grain/fruit as the harvest targets, total biomass yield is a key trait for forage crops, influenced by various phenotypic characteristics related to plant architecture, such as the number of tillers or branches, and leaf size (**Table** **5**). For annual silage crops including silage maize, forage oats, and sweet sorghum which are the primarily carbon and energy source for livestock, a balance is emphasized on the production of starchy grains and stem biomass.^[^ ^145^ ^]^ *Dry*, a plant‐specific NAC transcription factor, is a key domestication gene for the origin of sweet sorghum and plays a central role for biomass production and feed quality by regulating stem juiciness and sugar content.^[^ ^146^ ^]^ It functions as a crucial primary regulator for secondary cell wall biosynthesis in sorghum, and its defects result in altered cell morphology and cell wall composition.^[^ ^146^ ^]^ For perennial forage species like alfalfa and sheepgrass, the capability for regeneration post‐cutting or ‐grazing, the speed of spring regrowth, and the regulation of flowering time and fall dormancy (FD) are all important factors affecting biomass yield.^[^ ^147^ ^]^ Specifically, FD strongly influences the survival of forage crops in harsh winter conditions, while spring regrowth vigor directly impacts the yield in the early spring season.^[^ ^147^ ^]^

**Table 5:** Functional genes related to yield or quantity traits.

Type of Stress	Gene name	Species	Refs.
Yield	*MsD14*	*Medicago sativa*	[151]
	*MSAD_264347*	*Medicago sativa*	[154]
	*MsASMT1*	*Medicago sativa*	[155]
	*MsPAE12*	*Medicago sativa*	[156]
	*SPL13*	*Medicago sativa*	[157]
	*PvWOX3a*	*Panicum virgatum*	[158]
	*PvBiP2*	*Panicum virgatum*	[159]
	miR156	*Medicago sativa*	[150e]
	miR528	*Leymus chinensis*	[19]
	*Teosinte Branched 1*	*Leymus chinensis* / *Pancium virgatum*	[22, 160]
Forage quality	*CCoAOMT*	*Medicago sativa*	[161]
	*MtSGR*	*Medicago sativa*	[162]
	miR156	*Medicago sativa*	[163]
	*TT8*	*Medicago sativa*	[164]
	*HB12*	*Medicago sativa*	[164]
	*LpSGR*	*Lolium perenne*	[114]

Since some biomass‐related traits, such as FD, winter survival, and crown bud development, are phenotypically associated,^[^ ^148^ ^]^ it is critical to determine whether there is also a genetic linkage among these traits. Significant efforts have been made to map and develop markers for genetic loci controlling biomass in forage crops.^[^ ^149^ ^]^ Quantitative trait loci (QTL) analysis suggests that the genetic basis for flowering time and FD is likely unrelated.^[^ ^147^ ^]^ Therefore, it is possible to select superior alfalfa cultivars that are non‐dormant, winter‐hardy, and flower early in spring for enhanced biomass during the spring season.^[^ ^147^ ^]^

In addition to forward genetics and population‐based approaches, genetic engineering offers a promising strategy for enhancing forage crop biomass. Increasing plant height, the number of branches, and regrowth through the genetic engineering of morphogenetic regulators have proven effective for enhancing biomass in both leguminous and gramineous forage crops.^[^ ^150^ ^]^ For instance, silencing *MsD14*, a putative strigolactone receptor in alfalfa, results in increased shoot branching and forage biomass.^[^ ^151^ ^]^ In switchgrass, overexpression of *PvWOX3a*, a WUSCHEL‐related transcription factor, increases dry‐weight biomass by enhancing stem length, internode diameter, and leaf size.^[^ ^152^ ^]^ Additionally, a homozygous knockout mutation of monocot‐specific miRNA528 can improve growth rate and tiller number.^[^ ^19^ ^]^ Furthermore, genetic engineering of flowering time regulators has also been shown to enhance forage crop biomass.^[^ ^153^ ^]^

### 4.7 Forage Quality

Increasing digestibility has been a primary focus of research aimed at improving forage quality. High lignin content impedes carbohydrate degradation and digestibility, making it an undesirable trait in forage legumes.^[^ ^165^ ^]^ To reduce lignin content, many genes in the lignin biosynthetic pathway have been characterized and targeted through genetic engineering.^[^ ^166^ ^]^ However, reducing lignin content in plants often results in dwarfism and significant reductions in biomass yield. Therefore, large‐scale screening is necessary to identify individual germplasms that maintain an optimal lignin level without observable growth defects for commercial use.^[^ ^167^ ^]^ Work with *Brown midrib* (*BMR*) brachytic dwarf sorghum showed that enhanced feeding quality achieved by reducing the lignin content does not necessarily decrease drought tolerance and lodging resistance.^[^ ^168^ ^]^

The rapid degradation of forage crude proteins by rumen microbes can lead to pasture bloat, poor nutrient utilization by ruminant animals, and adverse effects on their health.^[^ ^43b^ ^]^ Proanthocyanidins (PAs) can complex with crude proteins, slowing down their metabolism by microorganisms. Forage crops with an appropriate amount of PA—typically between 2% and 4%—can exert beneficial effects on animal health.^[^ ^169^ ^]^ Foliar PA content varies significantly among forage crops. For example, alfalfa and *Trifolium repens* (white clover) contain negligible amounts of PA, while other legume forages, such as *O. viciifolia* (sainfoin), *L. corniculatus* (birdsfoot trefoil), and *Lotus pedunculatus* (big trefoil), as well as most gramineous forages, are less prone to causing pasture bloat.^[^ ^170^ ^]^ A large number of biosynthetic genes and regulatory factors involved in the PA biosynthesis pathway have been characterized in model plants and other plant species.^[^ ^170^ ^]^ PA accumulation can be effectively increased through the ectopic expression of these gene regulators, such as *MtPAR*, *TaMYB14*, and the maize *Lc*.^[^ ^171^ ^]^

## 5 Ploidy Breeding and Other Modern Breeding Techniques

### 5.1 Haploid Induction and Doubled Haploid Breeding

Many forage crops reproduce through out‐crossing and exhibit varying levels of heterozygosity. Doubled haploid (DH) plants, which contain two identical sets of homologous chromosomes, can be highly beneficial for forage crop breeding. Creating DH plants typically involves two essential steps: haploid induction (HI) followed by chromosome doubling. Traditional HI methods, such as microspore embryogenesis, rely on the in vitro culturing of haploid gametophytic tissue. However, this method is labor‐intensive and applicable only to a limited number of plant species due to its genotype dependency.^[^ ^172^ ^]^ An alternative approach involves cross‐pollination using haploid inducer lines, which can generate either maternal or paternal haploids in vivo, depending on the parent of origin of the retained haploid genome.^[^ ^172^ ^]^ The two most widely adopted HI systems are the maize Stock 6 maternal HI system and the Arabidopsis CENTROMERIC HISTONE3 (CENH3) modification system.^[^ ^173^ ^]^

**Figure 2:**
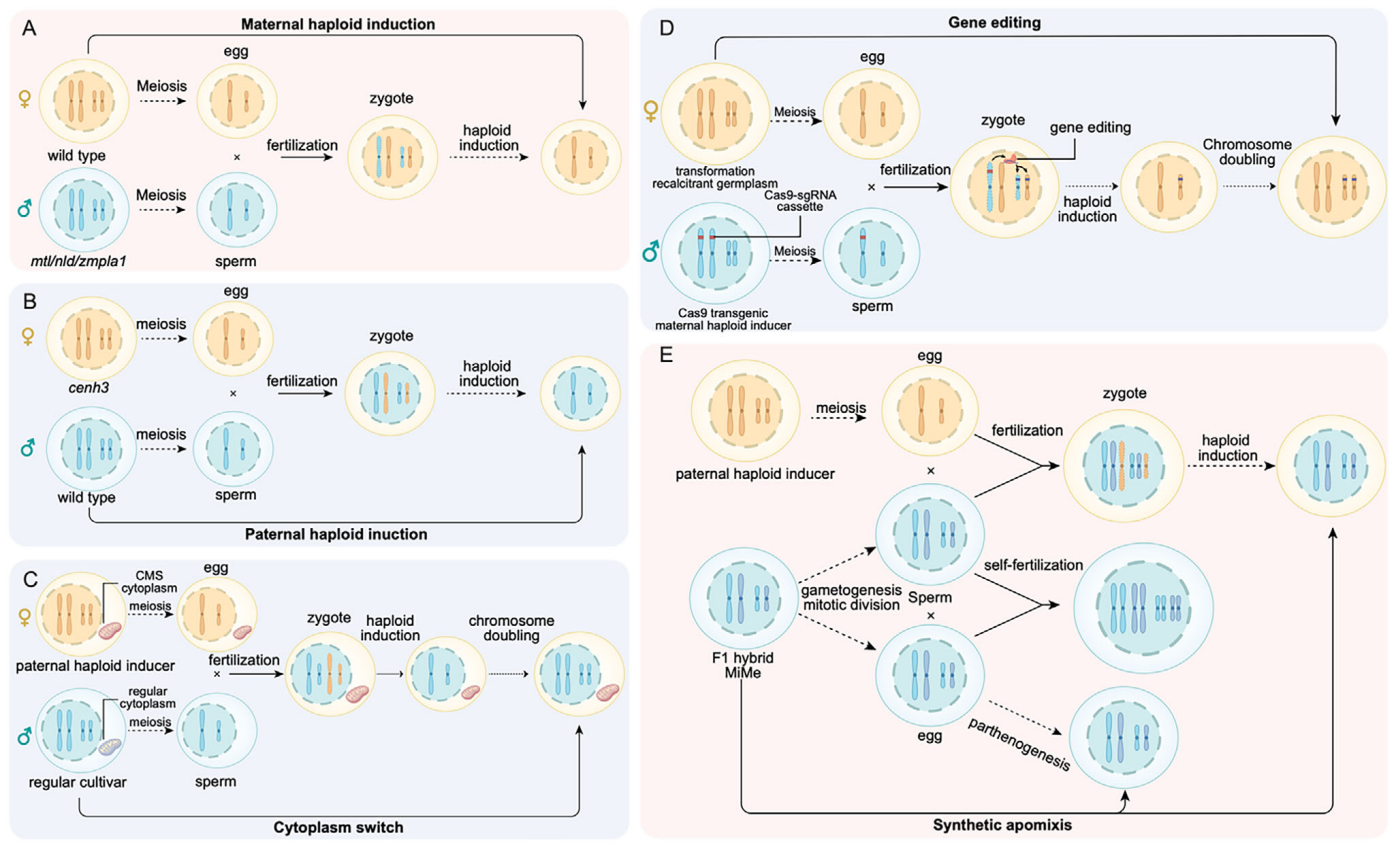
Haploid Induction and Its Application in Crop Breeding. A) Paternal Haploid Induction: Fertilization of egg cells carrying defective CENH3 results in the elimination of maternal chromosomes in the zygote. B) Maternal Haploid Induction: Fertilization by sperm with a defect in the *MTL/NLD/ZmPLA1* gene leads to the elimination of paternal chromosomes in the zygote. C) Cytoplasmic Replacement: Regular cytoplasm is replaced with male sterile cytoplasm in a cultivar of interest. The genetic background of the paternal haploid inducer is combined with male sterile cytoplasm, which serves as the maternal donor for crossing with a regular cultivar. After fertilization, the paternal chromosomes are retained, and the maternal male sterile cytoplasm is inherited. D) Gene Editing in Transformation‐Recalcitrant Germplasm: The CRISPR/Cas9 transgene cassette is integrated into the genome of the maternal haploid inducer, which is used to pollinate transformation‐recalcitrant germplasm. Transient expression of the Cas9 machinery in the zygote, prior to the elimination of the paternal genome, enables gene editing in the maternal genome. The resulting genetically edited, transgene‐free offspring is produced through maternal haploid induction. E) Maintaining Hybrid Vigor via Synthetic Apomixis: The MiMe background allows the generation of gametes with non‐reduced chromosomes. Clonal offspring of F1 hybrids can be produced either through parthenogenesis using the MiMe egg cell or through fertilization of egg cells from the paternal haploid inducer.

In the maize HI system, a spontaneous mutation in *MATRILINEAL (MTL)/PHOSPHOLIPASE A (ZmPLA1)/NOT LIKE DAD (NLD)* has been identified as a major QTL responsible for maternal haploid induction (**Figure** **2**A).^[^ ^174^ ^]^ Combining the *mtl*/*zmpla1*/*nld* mutation background with a second allele, the *zmdmp* mutation, further enhances HI efficiency.^[^ ^175^ ^]^ In contrast, the Arabidopsis *cenh3* null mutant, when complemented with a synthetic CENH3 protein featuring its amino‐terminal replaced by that of regular Histone 3 (GFP‐tailswap), exhibits significant defects in pollen development. This mutant can be pollinated with wild‐type pollen to generate paternal haploids (Figure 2B).^[^ ^173b^ ^]^

Using the aforementioned HI genes, in vivo haploid induction has been successfully applied in several plant species, including rice, wheat, tomato, onion, broccoli, and the model legume *Medicago truncatula*.^[^ ^176^ ^]^ There is significant potential for extending these strategies to other forage crops.

### 5.2 Other Applications of HI

Crossing between different plant varieties generates F1 hybrids that often exhibit superior traits compared to their parents. This phenomenon, known as hybrid vigor, has significantly influenced the seed production industry in major crops such as rice and maize, and it may also reshape the future of the forage crop industry.

Utilizing male sterility lines is crucial for eliminating the need for manual emasculation, thereby facilitating large‐scale field production of hybrid seeds. In the case of alfalfa, both nuclear and cytoplasmic male sterility (CMS) systems have been reported.^[^ ^177^ ^]^ Additionally, transgenic male‐sterile alfalfa has been developed.^[^ ^178^ ^]^ However, these male sterility traits have primarily been identified or created in a limited number of varieties.^[^ ^177^, ^178^ ^]^ To enable routine hybridization in forage crop breeding, it is essential to establish male sterility across diverse genetic backgrounds. The transfer of CMS to new varieties poses particular challenges, as it typically requires repeated backcrossing over several generations to achieve a nuclear–cytoplasm swap. Recently, researchers have demonstrated that nuclear–cytoplasm swaps can be significantly accelerated in both maize and broccoli using HI techniques through CENH3 modification (Figure 2C).^[^ ^176^, ^179^ ^]^

In addition to its applications in doubled haploid (DH) breeding and nuclear–cytoplasm swaps, HI technology facilitates crop breeding in many other ways. For instance, in both maize and Arabidopsis, it has been shown that the Cas9 editing machinery can be embedded in a haploid inducer line to generate targeted gene editing in transformation‐recalcitrant backgrounds through cross‐pollination (Figure 2D).^[^ ^180^ ^]^ This is made possible due to the transient zygotic state that exhibits Cas9 transgene expression prior to uniparental genome elimination.^[^ ^180^ ^]^ While there is significant potential for extending these strategies to other forage crops, one challenge in implementing the HI (haploid induction) technique in forage crops stems from the difficulty of generating suitable allele combinations in HI genes, particularly within high‐ploidy genetic backgrounds. Homozygous null mutations can lead to lethality, and the redundancy of multiple homologous gene copies often results in negligible disruptions to chromosome segregation. Consequently, developing techniques to precisely adjust allele dosage and produce a diverse range of hypomorphic mutants may be essential for identifying optimal HI parental lines.

### 5.3 Synthetic Apomixis

Many plant species, including various forage crops from the Poaceae family, can produce clonal offspring that carry identical genetic information to the parent. This phenomenon, known as apomixis, allows for the maintenance of elite cultivar phenotypes and ensures the faithful transmission of heterosis by preventing the recombination of genetic materials during meiosis.^[^ ^181^ ^]^

To harness this potential, a synthetic apomixis system called Mitosis instead of Meiosis (MiMe) has been engineered in planta by simultaneously mutating three key genes—*OSD1*, *SPO11*, and *REC8*—that are critical for meiosis.^[^ ^182^ ^]^ The MiMe plants produce clonal diploid gametes, generating tetraploid offspring upon self‐pollination. To obtain regular diploid clonal offspring, the MiMe system can be combined with the HI system to generate normal diploid progeny (Figure 2E).^[^ ^183^ ^]^

Alternatively, parthenogenesis—the development of embryos without fertilization—can be engineered within the MiMe plants through the egg cell‐specific expression of embryogenesis‐promoting regulators (Figure 2E).^[^ ^184^ ^]^ These synthetic apomictic strategies have effectively maintained hybrid vigor in self‐pollinated offspring.^[^ ^185^ ^]^ Unlike major staple crops, many forage crops are bred without the use of inbred lines due to their natural out‐crossing characteristics. Consequently, breeding materials of forage crops are often genetically heterogeneous. Maintaining hybrid vigor through synthetic apomixis is not only practically challenging due to the difficulties in creating high‐order mutations but also lacks strong motivation under current breeding strategies. However, in the long term, we anticipate that advancements in HI techniques may transform the way forage crops are bred, ultimately incorporating more sophisticated breeding approaches.

### 5.4 Plant Transformation and Genetic Engineering

Traditionally, improvements in forage crops were mainly achieved through recurrent crossing and selection, a time‐consuming process that heavily relies on the experience of breeders. Genetic engineering techniques, such as CRISPR/Cas9‐mediated gene editing, enable precise gene modifications to enhance desired traits, greatly facilitating crop improvement. The effectiveness of gene editing tools has been demonstrated in various cases, including increased tiller number and biomass in *Panicum virgatum* and *Leymus chinensis* through the knockout of the *TB1* (*Teosinte Branched 1*) gene, the generation of herbicide resistance in alfalfa via base editing of *ALS1* and *ALS2* (acetolactate synthase proteins), and the reduction of lignin content by mutating *COUMARATE 3‐HYDROXYLASE* in alfalfa.^[^ ^22^, ^160^, ^165^, ^186^ ^]^

**Figure 3:**
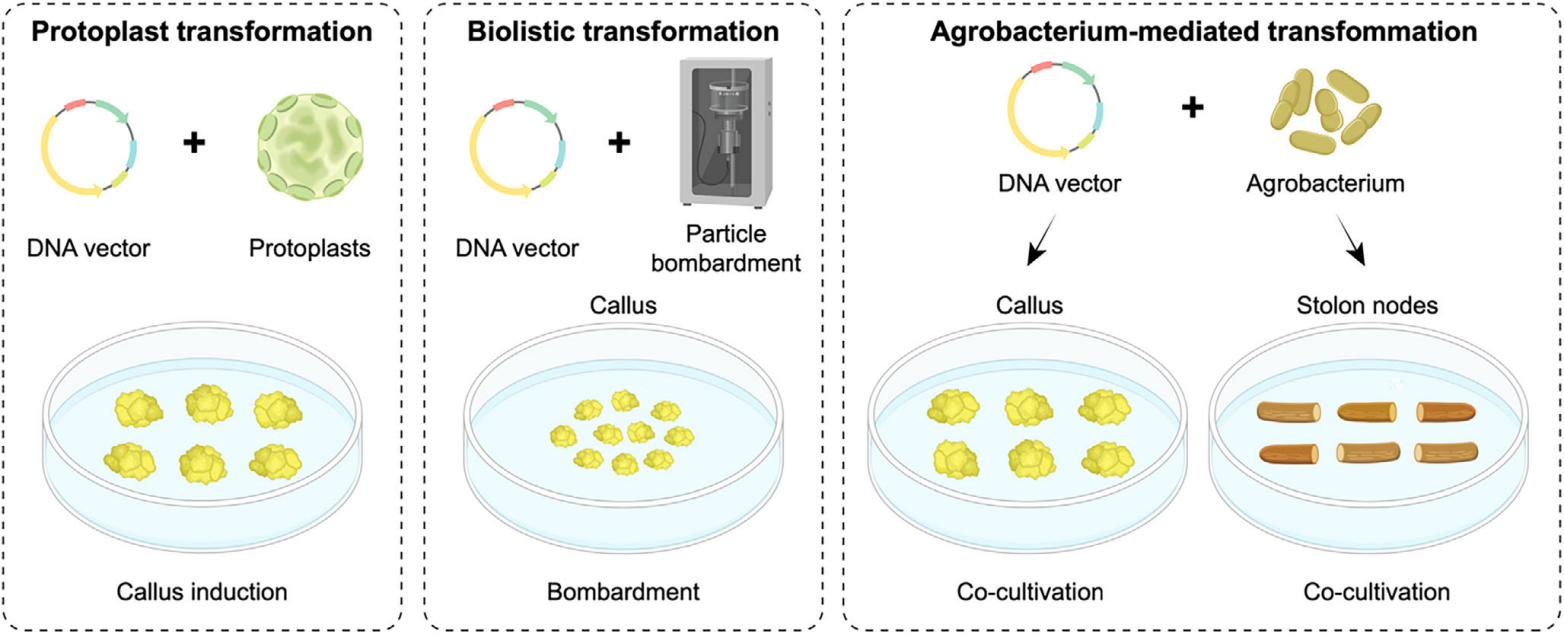
Transformation and Regeneration Methods for Forage Crop Genetic Engineering. Various tissue sources are used as explant donors. Transgenes are introduced via PEG treatment, particle bombardment, or Agrobacterium‐mediated transformation. Positive transformants are selected using different antibiotic markers, followed by plant regeneration.

The success of genetic engineering depends on the efficiency of transgene delivery and plant regeneration (**Figure** **3**). PEG‐mediated transgene delivery into protoplasts, followed by plant regeneration, has been reported for several *Festuca*, *Lolium*, and *Dactylis* species.^[^ ^187^ ^]^ A more common method for generating transgenic forage crops is Agrobacterium‐mediated transformation, established for *Medicago sativa*, *T. repens*, *Leymus chinensis*, *Panicum virgatum*, *Hordeum vulgare*, *Puccinellia tenuiflora*, and *Setaria viridis*.^[^ ^19^, ^22^, ^188^ ^]^ Another commonly used method is biolistic transformation (or particle bombardment) using high‐speed microparticles, which has also been reported for *Panicum virgatum*, *Cynodon dactylon*, and *L. perenne*.^[^ ^189^ ^]^

**Figure 4:**
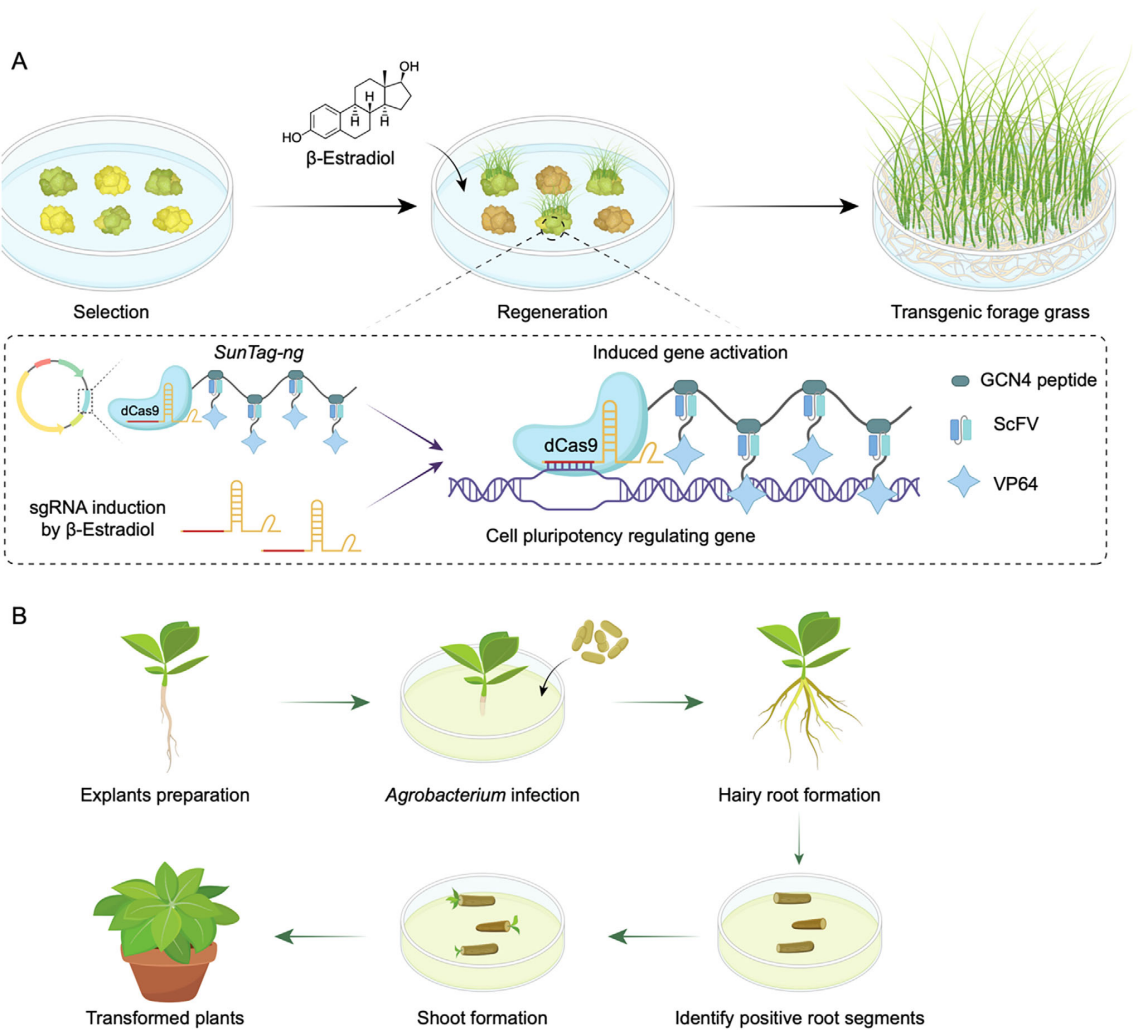
Novel strategies for enhanced regeneration and transformation in forage crops. A) Endogenous gene regulators of cell pluripotency are activated using CRISPR‐activation tools, such as the modified *SunTag* system. This approach involves inducing CRISPR activation modules with small molecule chemicals, enabling precise temporal control over the expression of regeneration‐promoting factors. B) Positive transgenic plants are regenerated directly from root segments following hairy root transformation. This method minimizes tissue culture requirements and circumvents genotype dependency.

Despite the growing use of genetic transformation tools in forage crop improvements, the generally low regeneration efficiency still limits the speed and scale of genetic engineering in these crops.^[^ ^2d^ ^]^ Regeneration efficiency varies greatly among individuals and is not only genotype‐dependent but also appears to be influenced by the type of donor explants used. The genetic basis underlying this variation in regeneration efficiency remains largely unknown. To overcome this bottleneck, the ectopic expression of morphogenic regulators known to induce cell pluripotency has been employed to enhance transformation and regeneration efficiency.^[^ ^190^ ^]^ In *Leymus chinensis*, ectopic expression of *TaWOX5* or activation of endogenous *LcBBM*/*LcLEC2* has been shown to improve regenerative capacity and transformation efficiency.^[^ ^22^, ^191^ ^]^ Novel strategies, such as the inducible activation of regeneration‐promoting morphogenetic regulators and methods that minimize or bypass tissue culture, such as GiFT (genotype‐independent fast transformation) and cut‐dip‐budding, will further expand the range of forage crop species and genotypes that are amenable to transformation (**Figure** **4**).^[^ ^191^, ^192^ ^]^

### 5.5 Designing by Molecular Modules

As most agronomic (economic) traits are controlled by multiple genes with “modular” characteristics, it is possible to develop new varieties through the assembly of multiple molecular modules.^[^ ^193^ ^]^ Owning to directional selection and improvement of target traits, molecular module design can significantly shorten the breeding cycles.^[^ ^194^ ^]^ In major crops, such as rice, several molecular modules have been characterized and were shown to regulate a variety of key agronomic traits, including cold tolerance,^[^ ^195^ ^]^ tillering and yield,^[^ ^196^ ^]^ hybrid vigor,^[^ ^197^ ^]^ seed development.^[^ ^198^ ^]^ Forage crop breeding can draw upon the successful experience of crop molecular module breeding to accelerate the development of new varieties.^[^ ^199^ ^]^

### 5.6 Genomic Selection

Genomic selection (GS) has emerged as a critical tool in the breeding of both animals and plants, with its significance increasingly recognized in the improvement of forage crops.^[^ ^2^, ^200^ ^]^ Unlike traditional approaches that focus on specific genetic loci, GS utilizes high‐density markers distributed across the entire genome to predict the breeding value of individuals, making it particularly effective for complex traits governed by multiple genes.^[^ ^201^ ^]^

The accuracy and potential of GS in predicting breeding values have been assessed in several forage crop species, including *Medicago sativa*, Triticale (× *Triticosecale* Wittmack), *L. perenne*, *Agropyron cristatum* (crested wheatgrass), and *Panicum maximum*.^[^ ^202^ ^]^ Notably, most classic statistical models are built on diploid genomes, while many forage crops are polyploid. Thus, integrating allele dosage effects into trait predictions is essential to enhance the efficiency of GS in forage crop breeding.

Additionally, traditional models based on best linear unbiased prediction and Bayesian methods have limitations in handling complex, non‐linear relationships between genotypes and phenotypes, particularly when working with high‐dimensional genomic data.^[^ ^203^ ^]^ To address these challenges, the application of machine learning techniques has emerged as a promising trend, offering increased accuracy in genomic selection.

## 6 Future Perspectives

### 6.1 Enhanced Utilization of Forage Germplasm Resources

Despite the vast reserves of global forage germplasm, in‐depth characterization has been inadequate, with only a fraction of these resources undergoing precise and limited trait assessments. There is an urgent need to establish a collaborative platform that integrates standardized genotypic and phenotypic evaluations for large‐scale, high‐throughput germplasm assessment. Leveraging genomics and phenomics approaches will enhance the efficiency of resource utilization, transforming germplasm resources into genetic strengths for forage breeding.

### 6.2 Cutting‐Edge Technologies for Genetic Improvements

At present, the application of genome editing technologies in forage crops is still in its infancy. Many advanced gene editing tools—such as multi‐gene editing, base editing, prime editing, and the targeted replacement or insertion of large DNA fragments—have yet to be fully implemented in forage crops. The editing efficiency is further challenged by the polyploid and heterozygous nature of many forage crops. High‐quality haplotype‐resolved genome assemblies are prerequisites for accurate and efficient gene editing, yet they have not been completed for the majority of forage crops. Therefore, enhancing the efficiency of gene editing in polyploid forage crops remains a key challenge for future research in forage genetic engineering.

### 6.3 Exploitation of the Microbiome for Forage Crop Breeding

Root‐associated microbiomes and endophytic fungi play crucial roles in plant growth, development, and environmental adaptation. Traditional domestication and breeding processes may have overlooked and even altered natural plant‐microbiome interactions, including the beneficial effects that enhance plant adaptability.^[^ ^204^ ^]^ In the future, we anticipate that forage crop breeders will develop more sustainable breeding and management strategies that combine the selection of superior plant traits with the customization of synthetic microbial communities.

### 6.4 Integrated and Intelligent Breeding for Leguminous and Gramineous Forage

Leguminous (e.g., alfalfa) and gramineous (e.g., ryegrass) forages exhibit distinct yet overlapping molecular regulatory networks, particularly in stress adaptation and nutrient utilization. While legumes leverage nitrogen‐fixing symbiosis for soil enrichment, gramineous species prioritize efficient nitrogen uptake and drought resilience. Notably, both families share conserved stress response pathways mediated by phytohormones such as abscisic acid (ABA) and jasmonic acid (JA). Aided by advanced breeding techniques, these interconnections offer untapped potential for cross‐species trait integration. For instance, transferring drought‐tolerance genes from ryegrass to alfalfa may enhance alfalfa performance under drought conditions. Such synergistic approaches promise to develop climate‐resilient, resource‐efficient forage varieties.

High biomass production remains the cornerstone and common goal of forage breeding, driven by optimized plant architecture that maximizes light interception and soil resource utilization across growth stages. This agronomically complex trait, encompassing leaf morphology, tillering capacity, and root depth, is governed by both genetic and environmental factors and modulated by management practices. The ideal architecture for both leguminous and gramineous forages combines upright growth for efficient light capture, sturdy stems for lodging resistance, and robust root networks for drought and grazing resilience. Precision irrigation, strategic cutting or grazing frequency, and intelligent nutrient management are critical to enhancing forage land stability and productivity.

The future of forage improvement lies in integrating cutting‐edge technologies to bridge genotype‐to‐phenotype gaps. Converging advances in high‐throughput phenotyping and expanding genomic databases will enable the rapid identification of trait‐associated genetic markers. Artificial intelligence (AI) is poised to revolutionize precision breeding, empowering breeders to predict optimal gene combinations for multi‐trait stacking, such as high biomass, nutrient efficiency, and resistance to abiotic and biotic stresses. Supported by precise gene‐editing tools like CRISPR‐Cas9, these innovations will accelerate the development of “smart” forage cultivars tailored for climate resilience, resource conservation, and sustainable livestock production. By unifying molecular insights, architectural optimization, and digital agriculture, integrated breeding systems will transform leguminous and gramineous forages into pillars of next‐generation agroecosystems.^[^ ^205^ ^]^

## Conflict of Interest

The authors declare no conflict of interest.
